# Complete chloroplast genome of Ulleung Island endemic basswood, *Tilia insularis* (Malvaceae), in Korea

**DOI:** 10.1080/23802359.2018.1473731

**Published:** 2018-05-18

**Authors:** Ji Young Yang, Woong Lee, Jae-Hong Pak, Seung-Chul Kim

**Affiliations:** a Research Institute for Dok-do and Ulleung-do Island, Kyungpook National University, Daegu, Republic of Korea;; b Department of Biological Sciences, Sungkyunkwan University, Suwon, Republic of Korea

**Keywords:** Chloroplast genome, Korean endemic basswood, *Tilia insularis*, Ulleung Island, Malvaceae

## Abstract

The first complete chloroplast genome sequences of Korean endemic basswood in Ulleung Island, *Tilia insularis*, were reported in this study. The *T. insularis* plastome was 162,565 bp long, with the large single-copy (LSC) region of 91,100 bp, the small single-copy (SSC) region of 20,381 bp, and two inverted repeat (IR) regions of 25,542 bp. The plastome contained 131 genes, including 86 protein-coding, eight ribosomal RNA, and 37 transfer RNA genes. The overall GC content was 36.5%. Phylogenetic analysis of 14 representative plastomes within the family Malvaceae suggests that *T. insularis* is closely related to other congeneric species in subfamily Tilioideae.

The basswood genus *Tilia* L. belongs to subfamily Tilioideae of Malvaceae and consists of 23 species widely distributed in the temperate northern hemisphere (Alverson et al. 1999; APG III 2009; Pigott 2012). *Tilia* is an ecologically important genus as one of the dominant deciduous tree species in the broad-leaved temperate forests and economically as useful timbers, honey resources, and ornamental trees (Pigott 2012; Cai et al. 2015; Bu and Park 2016). Of 23 species of *Tilia*, nine native and two cultivated species are known in Korea (Chung and Kim 1983; Kim and Chung 1986). Of nine native species, *T. insularis* Nakai is endemic to young volcanic Ulleung Island, which is located approximately 130 km east of the Korean peninsula (Nakai 1917; Kim 1985). Taxonomic status of *T. insularis* and its relationship to other species have long been controversial. For example, Yoon et al (2003) investigated the vegetative and reproductive morphology among *T. amurensis*, *T. insularis*, and *T. taquetii* in Korea and *T. japonica* in Japan, and concluded that the morphological differentiation among them were not sufficient enough to be recognized as distinct species. A similar morphological study between *T. amurensis* and *T. insularis* by Pigott (2008) also concluded that the two should be treated as conspecific. However, recent molecular phylogenetic study suggested that *T. insularis* is more closely related to *T. japonica* than *T. amurensis* (Bu and Park 2016). It is still required to determine the taxonomic status of *T. insularis* and its phylogenetic position (Chung and Kim 1983; Choi 2008). In this study, we sequenced the complete chloroplast genome of *T*. *insularis* and compared it to congeneric species.

Total DNA (Voucher specimen: KNU-Lee171010) was isolated using the DNeasy plant Mini Kit (Quiagen, Carlsbad, CA) and sequenced by the Illumina HiSeq 4000 (Illumina Inc., San Diego, CA). A total of 31,656,366 paired-end reads were obtained and assembled *de novo* with Velvet v. 1.2.10 using multiple *k*-mers (Zerbino and Birney 2008). The tRNAs were confirmed using tRNAsacn-SE (Lowe and Eddy 1997). The total plastome length of *T*. *insularis* (MH169579) was 162,565 bp, with large single copy (LSC; 91,100 bp), small single copy (SSC; 20,381 bp), and two inverted repeats (IRa and IRb; 25,542 bp each). The overall GC content was 36.5% (LSC, 34.2%; SSC, 31.0%; IRs, 43.0%) and the plastome contained 131 genes, including 86 protein-coding, eight rRNA, and 37 tRNA genes. A total of 17 genes were duplicated in the inverted repeat regions including seven tRNA, four rRNA, and six protein-coding genes. The complete *ycf*1 gene was included in the IR at the SSC/IRa junction and the *inf*A gene located in LSC became a pseudogene.

To confirm the phylogenetic position of *T*. *insularis*, 14 representative species of Malvaceae were aligned using MAFFT v.7 (Katoh and Standley 2013) and maximum likelihood (ML) analysis was conducted based on the concatenated 77 coding genes using IQ-TREE v.1.4.2 (Nguyen et al. 2015). The ML tree showed that *T*. *insularis* formed polytomy with *T. manshurica*, *T*. *paucicostata*, and *T*. *oliveri*.

**Figure 1.**
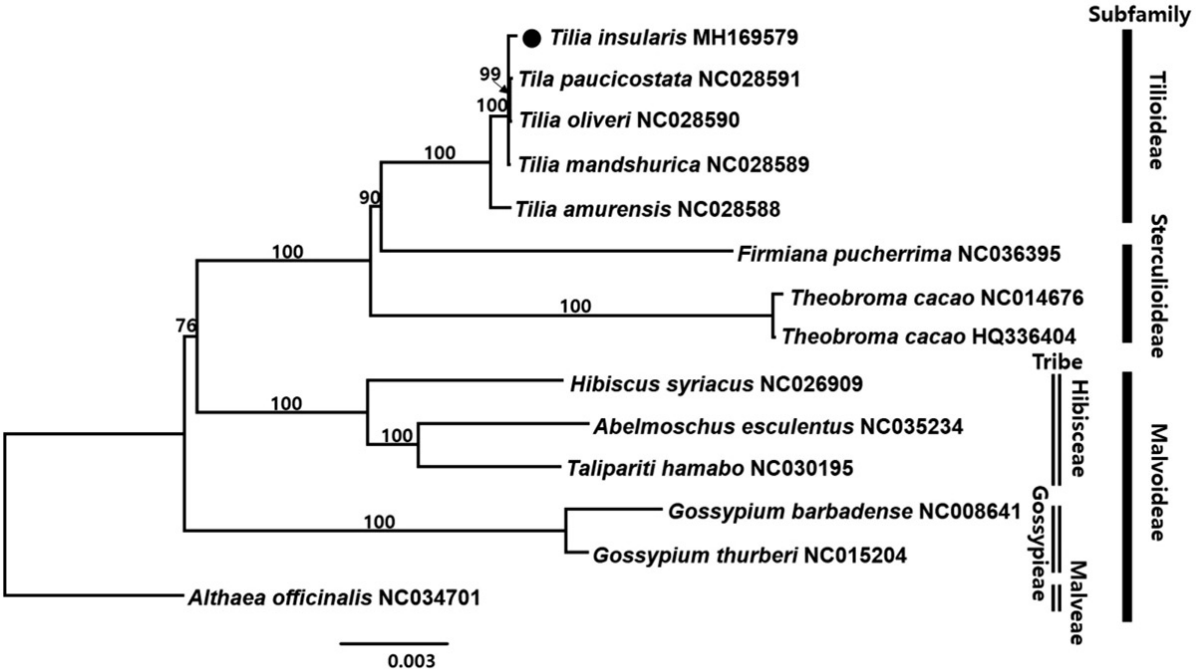
The maximum-likelihood (ML) tree based on 77 protein-coding genes in the 14 representative chloroplast genomes of Malvaceae. The bootstrap value based on 1000 replicates is shown on each node.
